# Predictors of health perceptions and health-promoting behaviors among Turkish prisoners: a cross-sectional study

**DOI:** 10.1186/s12889-025-24189-8

**Published:** 2025-10-06

**Authors:** Şadiye ÖZTÜRK, Nilay ERCAN-ŞAHİN

**Affiliations:** 1 Public Health Nursing Department, Faculty of Health Sciences, Eskişehir Osmangazi University, Eskişehir, Turkey; 2 Public Health Nursing Department, Nursing Faculty, Hacettepe University, Ankara, Turkey

**Keywords:** Correctional health nursing, Health lifestyle behaviors, Health perception, Prisoners

## Abstract

**Background:**

Prisoners are considered a vulnerable group in terms of health risks and limited access to health services. This study aimed to determine the health perceptions and health-promoting behaviors of Turkish prisoners, examine the relationship between these factors, and identify the predictors of health perceptions and health-promoting behaviors as a cross-sectional design.

**Methods:**

This cross-sectional study was conducted between November 2023 and January 2024 with 234 prisoners convicted of criminal offenses in two open penal institutions in Eskişehir, Turkey. Data were collected using a sociodemographic information form, the Health Perception Scale, and the Healthy Lifestyle Behaviors Scale II. Statistical analyses included Independent Samples t-test, One-way ANOVA, Pearson correlation analysis, and multiple linear regression analysis to identify the predictors of health perceptions and health-promoting behaviors.

**Results:**

The mean score of health perception among the prisoners was 51.43 ± 7.66, and the mean score of healthy lifestyle behaviors was 130.41 ± 26.90. A positive but weak correlation was found between health perception and healthy lifestyle behaviors (*r* = .319; *p* < .001).

**Conclusions:**

The findings highlight the importance of evaluating prisoners’ health perceptions and health-related behaviors. Prison nurses are recommended to routinely assess these factors and implement targeted interventions to support healthy lifestyle changes in prison populations.

## BACKGROUND

The number of prisoners and detainees worldwide continues to increase. According to the World Prison Population List, there were more than 11.5 million prisoners and detainees globally in 2021 [1]. Similarly, the Annual Penal Statistics of the Council of Europe reported that there were 272.115 prisoners and detainees in Europe in 2021, with Turkey and Russia being the countries with the highest prison populations [2].

During their incarceration, prisoners experience severe restrictions on their freedom of movement and must carry out their eating, drinking, sleeping, and daily activities collectively within the confines of the prison [3]. This situation negatively affects their physical and mental health. Factors such as inadequate nutrition, insufficient ventilation, lack of hygiene, loneliness, lack of privacy, and the negative attitudes of other prisoners make prisoners particularly vulnerable in terms of their health and care needs [4]. Recent evidence highlights the elevated burden of physical and mental health conditions among prisoners. A 2024 umbrella review reported that 11.4% of inmates experienced major depression, 9.8% had post-traumatic stress disorder, and 3.7% had psychotic disorders. Upon entry, 23.8% met criteria for alcohol use disorder and 38.9% for drug use disorder, with high comorbidity. Additionally, 17.7% tested positive for hepatitis C, while rates of hepatitis B, HIV, and tuberculosis were also notable [5]. Supporting this, a study in two Turkish prisons found 4.7% of inmates were HBsAg positive, 16.9% showed markers of past HBV infection, and 0.5% were anti-HCV positive, underscoring the need for infection screening and prevention in correctional settings [6].

Prisoners’ health perception is a crucial concept that provides insight into how prisoners evaluate their overall health status [7]. Compared to the general population, prisoners have been found to have lower health perceptions. In a study by Nobile et al., 60% of prisoners reported that their health had deteriorated during their incarceration [8]. Research has shown that factors such as age, educational level, presence of chronic illness, and depressive symptoms significantly influence prisoners’ health perceptions [7–10]. For instance, older prisoners tend to have lower health perceptions, which has been associated with decreased physical activity levels and an increased risk of cardiovascular diseases [10]. Conversely, prisoners with higher educational levels have been found to exhibit better health perceptions [7, 8].

Health perception plays a crucial role in individuals’ adoption and maintenance of healthy lifestyle behaviors [11]. Prisoners with higher health perceptions have been found to engage in healthier lifestyle behaviors more frequently [12]. Under prison conditions, these behaviors have been shown to be influenced by factors such as educational level, occupation, place of residence, and previous incarceration experience [13]. In this context, healthy lifestyle behaviors encompass a range of domains that are particularly meaningful in prison settings. These include spiritual growth, which may serve as a psychological coping strategy in restrictive environments; health responsibility, reflecting inmates’ awareness and engagement in their own care; physical activity, often limited by institutional regulations; nutrition, which may be compromised by standardized meal plans; interpersonal relations, shaped by forced cohabitation and limited privacy; and stress management, which is critical given the high-stress nature of incarceration. These sub-dimensions help capture the multifaceted nature of health behavior within correctional institutions.

The successful reintegration of prisoners into society in a healthy manner is essential for building healthier communities [14]. Health is broadly defined by the World Health Organization as a state of complete physical, mental, and social well-being, not merely the absence of disease [15]. Health promotion refers to the process of enabling people to increase control over, and to improve, their health [16]. In prison settings health promotion may involve education, prevention, and behavior change initiatives tailored to prisoners’ unique vulnerabilities [17]. For instance a study conducted by Tesler et al. demonstrated that engagement in a health promotion program was positively correlated with increased levels of physical activity and improved subjective health perceptions [18]. The World Health Organization also emphasized in the Ottawa Charter that prisons are among the key settings where health promotion should be prioritized. In this context, preventing infectious diseases, reducing health-related risks, providing health education and counseling, ensuring hygienic environments, preserving individuality, and preventing illicit drug use should be among the primary objectives of healthcare services in prisons [19].

Prison nurses play a crucial role in the provision of healthcare services within correctional facilities. They have significant responsibilities, including identifying existing health problems among prisoners, monitoring their health status, and providing health education [14]. In this process, assessing prisoners’ health perceptions and healthy lifestyle behaviors is essential for planning and improving the healthcare services provided to them [20].

Although research on prisoners’ health perceptions and healthy lifestyle behaviors is limited in the international literature, existing studies do address these topics [7, 20, 21]. However, most of these studies primarily focus on how prisoners perceive their health or on specific health issues. Therefore, this study aims to determine prisoners’ health perceptions and healthy lifestyle behaviors, as well as to examine the relationship between them. By identifying these aspects, the study is expected to guide the planning and implementation of nursing interventions aimed at improving prisoners’ health.

## Methods

This cross-sectional descriptive study was conducted between November 2023 - January 2024 in two open penal institutions in Eskişehir, Türkiye. Using a cross-sectional descriptive design, this study aimed to determine the health perceptions and health-promoting behaviors of prisoners, examine the relationship between these variables, and identify the predictors of both health perceptions and health-promoting behaviors. Specifically, the study sought to answer the following questions: What is the current mean score of prisoners’ health perceptions and health-promoting behaviors? Is there a statistically significant relationship between these two constructs? And what are the predictors of health perception and healthy lifestyle behavior among prisoners?

A cross-sectional design was deemed appropriate as it allows for the assessment of participants’ perceptions and behaviors at a single point in time without follow-up, which aligns with the study’s descriptive aims.

### Setting and sample

The study was conducted at the Eskişehir No. 1 and No. 2 Open Penitentiary Institutions, affiliated with the Directorate of Prisons and Detention Houses in Turkey. At the time of data collection, the total prisoner population across these institutions was 591, which constituted the target population (universe) of the study. To determine a representative sample from this population, the known population sample size formula was used, based on a 95% confidence level and 5% margin of error. As a result, the minimum required sample size was calculated to be 234.

**Fig. 1:**
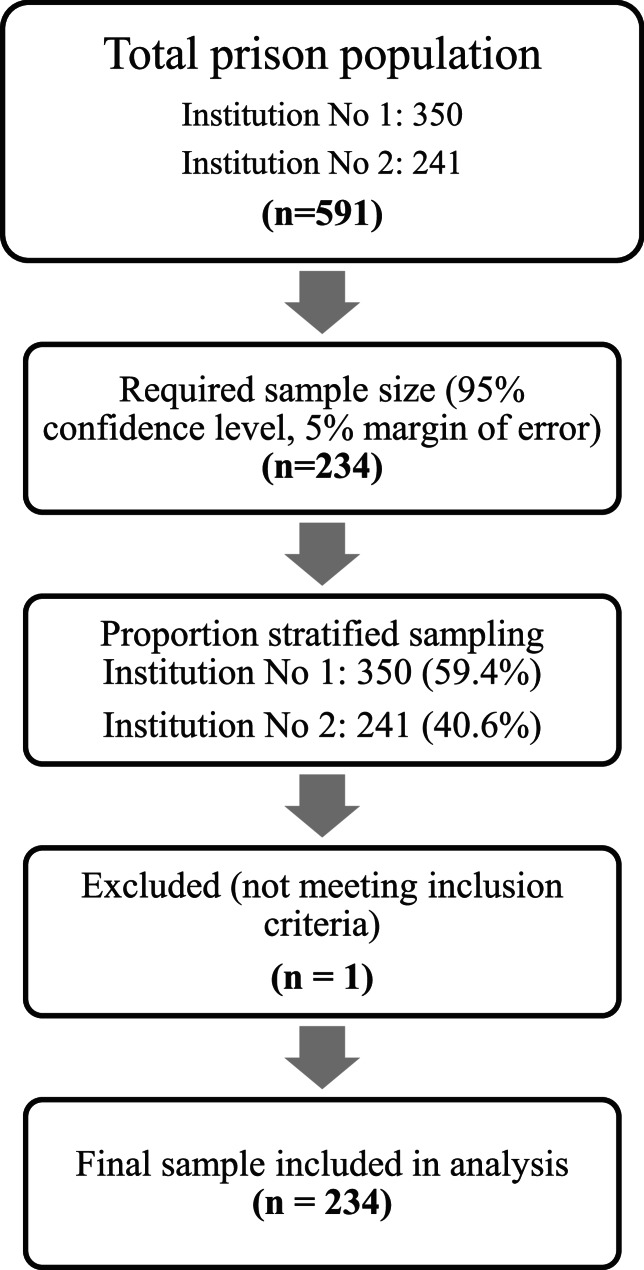
Flow Diagram of Participant Selection and Inclusion

In order to ensure proportional representation of the prisoners from both institutions, proportional stratified sampling was employed. According to this method, 139 participants were selected from No. 1 Penal Institution and 95 participants from No. 2 Penal Institution, corresponding to their respective population weights (59.2% and 40.8%). A flow diagram illustrating the participant selection process is presented in Fig. 1.

Inclusion criteria were being a convicted inmate residing in one of the two open penal institutions and voluntarily agreeing to participate in the study. Exclusion criteria included individuals convicted of terrorism-related offenses, based on the official permission obtained from the Ministry of Justice, and individuals who were unable to complete the questionnaire due to cognitive limitations. Accordingly, one inmate was excluded.

Two separate post-hoc power analyses were conducted in the study. The first, based on an independent samples t-test comparing Health Perception Scale scores by physical activity status, yielded an achieved statistical power of 0.80, indicating sufficient power to detect medium-sized effects. The second analysis, conducted on the association between Healthy Lifestyle Behavior Scale scores and Health Perception, resulted in an achieved power of 0.99, reflecting a very high likelihood of detecting a true effect and indicating strong reliability of the findings.

Participants were typically housed in shared wards, with capacities ranging from small 4-person units to larger wards accommodating up to 54 person. They had access to communal areas such as workshops, conference halls, dining halls, and hygiene facilities. Data collection was conducted in these common spaces, reflecting the daily environmental conditions experienced by participants.

### Data collection

#### Data collection tools

The Sociodemographic Information Form, Health Perception Scale (HPS) and Healthy Lifestyle Behaviors Scale II (HLBS-II) were used by the researchers within the scope of the data collection.

#### Sociodemographic information form

This form consists of a total of 21 questions related to sociodemographic information about the prisoners and their healthy lifestyle behaviors. The form includes questions regarding age, educational status, marital status, parenthood, the duration of time spent and to be spent in prison, prison conditions, communication with family, the prisoner’s health status, health promotion, and hygiene habits [22–24]. As part of this form, participants were asked binary (Yes/No) questions regarding their health behaviors. Specifically, regular physical activity was assessed by the question: “Do you engage in regular physical activity?” and dietary habits were assessed with the question: “Do you think you eat a healthy diet?” Other health-related behaviors, such as smoking, tooth brushing, showering frequency, and sharing personal items, were also assessed using binary (Yes/No) self-report questions.

#### Health perception scale (HPS)

The Health Perception Scale was developed by Diamond et al. (2007) and its Turkish validity and reliability were established by Kadıoğlu and Yıldız (2012). The scale consists of 15 items and four sub-dimensions: locus of control, certainty, importance of health, and self-awareness. It is rated on a five-point Likert scale [25, 26].

Items 1, 5, 9, 10, 11, and 14 are positively worded and scored as follows: Strongly agree = 5, Agree = 4, Neutral = 3, Disagree = 2, Strongly disagree = 1. In contrast, items 2, 3, 4, 6, 7, 8, 12, 13, and 15 are negatively worded and reverse-scored. Higher scores on the scale indicate a more positive health perception. Since this scale do not have standard cut-off points, mean scores and their distribution were used to interpret the findings.

The Cronbach’s Alpha reliability coefficient for the overall scale was reported as 0.70, with sub-dimension reliability coefficients of 0.67, 0.73, 0.54, and 0.53, respectively [26]. In the present study, the Cronbach’s Alpha coefficient for the overall scale was 0.63, while the reliability coefficients for the sub-dimensions were 0.73, 0.71, 0.51, and 0.54, respectively.

#### Healthy life style behavior scale II (HLBS-II)

The Healthy Life Style Behavior Scale II was developed by Walker et al. (1996), and its Turkish validity and reliability study was conducted by Bahar et al. (2008). The scale consists of 52 items and six sub-dimensions: spiritual growth, health responsibility, physical activity, nutrition, interpersonal relations, and stress management [27].

It is rated on a four-point Likert scale: Never (1), Sometimes (2), Often (3), Regularly (4). Higher scores indicate a higher frequency of engaging in health-promoting behaviors. Since this scale do not have standard cut-off points, mean scores and their distribution were used to interpret the findings.

The Cronbach’s Alpha reliability coefficient for the overall scale was reported as 0.92, with sub-dimension coefficients of 0.77, 0.79, 0.68, 0.79, 0.80, and 0.64, respectively [27]. In the present study, the Cronbach’s Alpha coefficient for the overall scale was 0.95, and the sub-dimension reliability coefficients were 0.80, 0.82, 0.71, 0.87, 0.82, and 0.74, respectively.

### Procedure

Data were collected between November 2023 and January 2024 from two open penal institutions located in Eskişehir, Türkiye. In total, 591 inmates constituted the study population. Due to institutional constraints, random sampling was not feasible; instead, proportional stratified sampling was used to ensure representative distribution based on the population size of each institution. The calculated sample size was 234, and participants were selected accordingly.

In the first institution, the researcher visited the site on three separate occasions to reach the targeted sample size. During the first visit, inmates who did not meet the inclusion criteria (individuals convicted of terrorism-related crimes, as per institutional directive) were identified. In subsequent visits, prisoners were invited to participate through institutional announcements made via loudspeakers or through direct contact in prison workshops. Those who volunteered were gathered in communal areas such as conference halls or workshops, where they completed the questionnaires independently.

In the second institution, the researcher visited the site twice. Similar procedures were followed, with announcements made by prison staff and face-to-face invitations extended in work areas. Questionnaires were distributed and collected by the primary researcher. Institutional personnel such as psychologists, administrative staff, and correctional officers provided logistical support during data collection to ensure a smooth process. For participants with reading or vision difficulties, the researcher read the questions aloud and recorded their responses to ensure data integrity and inclusivity. To minimize response bias, participation was voluntary and anonymous, and no identifying information was collected. The researcher emphasized confidentiality and encouraged honest responses. For participants with reading or vision difficulties, the questionnaire was administered orally by the researcher in a one-on-one setting to maintain data quality and inclusivity.

### Data analysis

The collected data were analyzed using Statistical Package for the Social Sciences (SPSS) version 27.0. To assess the normality of the data distribution, Skewness and Kurtosis values, Kolmogorov-Smirnov significance value, Q-Q plot, Box-plot, and histogram were examined (Table 1).

**Table 1:** Normality test results

Variable	Skewness	Kurtosis	K-S Statistic	*p*-value	Interpretation
Health Perception Total Score	0.433	0.216	0.052	0.200	Normal distribution
HLBS-II Total Score	−0.227	0.195	0.045	0.200	Normal distribution

Categorical variables (e.g., age groups, education level, number of inmates per ward, smoking status, physical activity) were summarized as frequencies and percentages. Continuous variables such as health perception score and HLBS-II total scores were presented as means and standard deviations. Since the scores showed normal distribution, parametric tests were used in the analysis.

Descriptive statistics were presented as mean, standard deviation, frequency, percentage, median, and minimum-maximum values. Independent Samples t-test was used to compare the means between two groups. One-way ANOVA was applied to compare means across more than two groups. In cases where significant differences were found between groups, post-hoc comparisons were made using Scheffe and LSD tests. Pearson correlation analysis was conducted to assess relationships between variables and multiple linear regression analysis to identify the predictors of health perceptions and health-promoting behaviors. Prior to regression, bivariate analyses were used to determine candidate variables for inclusion in the model. Only those variables found to be statistically significant in bivariate tests (*p* <.05) were included as independent variables in the regression models.

## RESULTS

In accordance with the research objectives, the findings are presented in response to the four main research questions of this study: (1) the level of prisoners’ health perceptions, (2) the level of their health-promoting behaviors, (3) the relationship between these two variables, and (4) the predictors of health perceptions and healthy lifestyle behaviors.

As indicated in Table 2, a statistically significant discrepancy was identified between the educational attainment of the prisoner population and the total score of the HPS and the total score of the HLBS-II. Post-hoc analysis using the LCD test indicated that prisoners with secondary and higher education levels had significantly higher HPS and HLBS-II scores compared to those with primary education and below.

**Table 2:** Score distribution of HPS and HLBS-II according to some characteristics of prisoners

Variables	*n*(%)	HPS Total Score	95% CI Lower	95% CI Upper	Statistical Analysis	HLBS-II Total Score	95% CI Lower	95% CI Upper		Statistical Analysis
		Mean ± SD			t/F; *p*	Mean ± SD				t/F; *p*
Age										
18–25 26–35 36–45 46 and above	28(12.0) 88(37.6) 75(32.1) 43(18.4)	50.71 ± 5.87 51.25 ± 7.12 51.09 ± 7.54 52.88 ± 9.75	48.436 49.740 49.358 49.880	52.992 52.759 52.828 55.887	0.659 0.578	123.96 ± 27.33 129.39 ± 27.80 135.02 ± 23.39 128.65 ± 29.96	113.363 123.506 129.644 119.430		134.565 135.289 140.408 137.872	1.382 0.249
Level of education										
Primary and below Secondary education Higher education	118(50.4) 90(38.5) 26(11.1)	49.98 ± 7.28 52.66 ± 7.40 53.76 ± 9.06	48.654 51.116 50.109	51.311 54.217 57.429	4.630 0.011*	126.12 ± 27.41 133.56 ± 25.69 138.96 ± 26.06	121.129 128.185 128.432		131.125 138.947 149.490	3.502 0.032*
Number of people in a ward										
≤ 10 people 11–20 people 21–30 people ≥ 31 people	45(19.2) 81(34.6) 78(33.3) 30(12.8)	50.93 ± 7.30 51.66 ± 7.23 51.05 ± 8.17 52.56 ± 8.16	48.738 50.067 49.208 49.519	53.128 53.265 52.893 55.614	0.370 0.775	132.26 ± 24.15 136.82 ± 25.42 124.60 ± 27.54 125.43 ± 29.91	125.009 131.205 118.392 114.261		139.524 142.449 130.812 136.604	3.253 0.023^**^
Time spent in prison										
1 year and less 2–3 years 4–5 years 6 years and above	84(35.9) 41(17.5) 40(17.1) 69(29.5)	50.34 ± 6.92 53.41 ± 8.43 52.87 ± 7.33 50.75 ± 8.01	48.842 50.751 50.528 48.828	51.847 56.078 55.222 52.679	2.165 0.093	129.08 ± 27.81 135.24 ± 23.30 131.65 ± 23.35 128.44 ± 29.70	123.048 127.886 124.181 121.313		135.118 142.601 139.118 135.585	0.657 0.579
Presence of a diagnosed chronic disease										
No Yes	171(73.1) 63(26.9)	51.22 ± 6.98 52.00 ± 9.28	−3.326	1.782	−0.600 0.550	130.24 ± 25.73 130.87 ± 30.05	−8.455		7.200	− 0.158 0.875
Physical activity status										
No Yes	115(49.1) 119(50.9)	50.18 ± 6.75 52.64 ± 8.28	−4.416	−5.525	−2.488 0.014	121.77 ± 28.33 138.76 ± 22.59	−23.607		−10.374	−5.061 0.000
Healthy nutrition status										
No Yes	138(59.0) 96(41.0)	51.40 ± 7.48 51.47 ± 7.93	−2.083	1.936	−0.072 0.943	128.93 ± 28.21 132.54 ± 24.87	−10.651		3.437	−1.009 0.314
Smoking status										
No Yes	51(21.8) 183(78.2)	54.62 ± 8.36 50.54 ± 7.22	1.745	6.416	3.442 0.001	136.29 ± 28.13 128.77 ± 26.39	−0.836		15.872	1.773 0.078
Tooth brushing status										
No brushing At least 1 time a day	46(19.7) 188(78.2)	48.97 ± 7.36 52.03 ± 7.62	−5.515	−0.602	−2.454 0.015	117.10 ± 30.33 133.67 ± 25.02	−26.220		−6.902	−3.429 0.001
Frequency of showering										
Irregular At least 1 a day 1–2 per week Every two days	8(3.4) 153(65.4) 21(9.0) 52(22.2)	46.87 ± 4.05 51.19 ± 7.47 49.66 ± 6.66 53.55 ± 8.52	43.488 50.002 51.184 46.632	50.261 52.389 55.931 52.700	2.760 0.043^*^	111.87 ± 29.08 132.98 ± 26.74 118.76 ± 28.93 130.40 ± 24.49	87.560 128.714 123.584 105.592		136.189 137.259 137.223 131.931	3.130 0.026^*^
Sharing personal belongings										
No Yes	226(96.6) 8(3.4)	51.63 ± 7.65 45.75 ± 5.54	0.499	11.274	2.153 0.032	131.42 ± 26.28 102.00 ± 30.54	10.693		48.147	3.095 0.002
Total		51.43 ± 7.66				130.41 ± 26.90				

*LSD test was performed. **Scheffe test was performed. F: Anova t: t test

It was found that there was a statistically significant difference between the number of people staying in the ward and the total score of the HLBS-II. Post-hoc analysis using the Scheffe test indicated that this difference was due to the fact that the total score of the HLBS-II of the prisoners staying in the 21–30 person wards was lower than the prisoners staying in the 11–20 person wards (Table 2).

A statistically significant difference was found between the frequency of showering and both the HPS and the HLBS-II total scores. Post hoc analysis using the LCD test indicated that that prisoners who reported showering once or twice per week had significantly higher HPS and HLBS-II scores than those who showered irregularly. Moreover, prisoners who showered every two days also had higher HLBS-II scores compared to those with irregular showering habits.

The distribution of key health-related behaviors among the participants is as follows: Among the prisoners, 78.2% reported smoking, 41.0% stated they followed a healthy diet, and 50.9% engaged in regular physical activity. Additionally, 26.9% of the participants had at least one chronic illness, 78.2% reported brushing their teeth regularly, and 3.4% reported sharing personal belongings. It was found that there was a significant difference between regular physical activity, smoking, regular tooth brushing, and use of shared personal items and total score of HPS (Table 2). A significant difference was found between the total HLBS-II score and certain health behaviors, including regular physical activity, regular tooth brushing, and the use of shared personal items (Table 2).

To address the first and second research questions, the overall scores and sub-dimensions of the scales were examined. The distribution of prisoners’ scores on the health perception and healthy lifestyle behaviors scales and their sub-dimensions are presented in Table 3. The prisoners received a mean score of 51.43 ± 7.66 (min. 34.00 – max. 75.00) on the health perception scale and a mean score of 130.41 ± 26.90 (min. 52.00 – max. 205.00) on the healthy lifestyle behaviors scale. Since there are no validated cut-off points for the scales used, only mean scores were reported without categorizing participants into levels.

**Table 3:** Distribution of the relationship between the HLBS II and HPS total scores of prisoners

Scales and sub-dimensions	1	1.1	1.2	1.3	1.4	2	2.1	2.2	2.3	2.4	2.5	2.6
1. HPS total score	1	-	-	-	-	-	-	-	-	-	-	-
1.1. Control center	0.769	1	-	-	-	-	-	-	-	-	-	-
1.2. Certainty	0.624	0.426	1	-	-	-	-	-	-	-	-	-
1.3. The importance of health	0.302	−0.123	−0.247	1	-	-	-	-	-	-	-	-
1.4. Self-awareness	0.33	0.191	0.132	0.296	1	-	-	-	-	-	-	-
2. HLBS II total score	0.319	0.148	0.134	0.295	0.871	1	-	-	-	-	-	-
2.1. Responsibility for health	0.189	0.068	0.044	0.248	0.648	0.836	1	-	-	-	-	-
2.2. Physical activity	0.275	0.109	0.111	0.241	0.553	0.785	0.55	1	-	-	-	-
2.3. Nutrition	0.267	0.131	0.169	0.187	0.562	0.796	0.624	0.683	1	-	-	-
2.4. Spiritual development	0.33	0.191	0.132	0.296	0.17	0.871	0.648	0.553	0.562	1	-	-
2.5. Interpersonal relationships	0.273	0.123	0.091	0.271	0.836	0.861	0.714	0.494	0.542	0.836	1	-
2.6. Stress management	0.262	0.113	0.136	0.222	0.719	0.876	0.66	0.693	0.656	0.719	0.698	1

Regarding the third research question, Pearson correlation analysis revealed that there is a moderate positive relationship between health perception and healthy lifestyle behaviors (*r* = 0.319; *p* < 0.001). There is a positive relationship between health perception and health responsibility (*r* = 0.189; weak), physical activity level (*r* = 0.275; weak), nutrition perception (*r* = 0.267; weak), spiritual development (*r* = 0.330; moderate), interpersonal relationships (*r* = 0.273; weak) and stress management (*r* = 0.262; weak) (*p* < 0.001 for each). There is a positive relationship between prisoners’ healthy lifestyle behaviors and center of control (*r* =0.148; weak), certainty (*r* =0.134; weak), importance of health (*r* =0.295; weak) and self-awareness (*r* =0.871; strong) (*p* <0.05 for each) (Table 3).

The significant variables that were effective on the health perception of prisoners in binary analyses were included in the model and the variables in the model formed as a result of multiple linear regression analysis were found to be effective on the health perception of prisoners (F (4.233) = 7.090; *p* < 0.001). The explanatory power (R^2^) of the four variables in the model on health awareness is 11%. When the coefficients of the model were analyzed, the health perception of the prisoners decreased by 6.016 points in case of shared use of personal belongings, by 3.329 points in case of smoking, by 2.085 points in case of having primary education level or less, and increased by 2.314 points in case of regular physical activity (Table 4).

**Table 4:** Results of multiple linear regression analysis for the prediction of prisoners’ HPS total scores

HPS Scale	Unstandardized Coefficients		Standardized Coefficients	t	*p*	95% CI		Collinearity Statistics	
	B	SH	B			Upper	Lower	Tolerance	VIF
(Fixed)	54.120	1.191		45.427	0.000				
Smoking	−3.329	1.192	−0.180	−2.793	0.006	−5.678	−0.981	0.938	1.067
Physical activity	2.314	0.956	0.151	2.420	0.016	0.430	4.198	0.994	1.006
Sharing personal belongings	−6.016	2.628	−0.143	− 0.2.289	0.023	−11.195	−0.837	0.995	1.005
Primary education level and below*	−2.085	0.984	−0.136	−2.119	0.035	−4.023	−0.147	0.938	1.066

R^2^ = 0.110; Adjusted R^2^ = 0.095; Durbin-Watson = 2.334; F (4.233) = 7.090; *p* <0.001

*Reference categories: Secondary education

The significant variables that were effective on the healthy lifestyle behaviors of prisoners in binary analyses were included in the model and the variables in the model formed as a result of multiple linear regression analysis were found to be effective on the healthy lifestyle behaviors of prisoners (F (4.233) = 13.909; *p* < 0.001). The explanatory power (R^2^) of the four variables in the model on healthy life behaviors is 19.5%. When the coefficients of the model were examined, the healthy living behaviors of the prisoners decreased by 32,041 points in the case of shared use of personal belongings, 7.738 points in the case of having primary education level or less, 16.763 points in the case of regular physical activity, and 9,832 points in the case of staying in wards for 11–20 people (Table 5).

**Table 5:** Multiple linear regression analysis results on the prediction of HLBS II of prisoners

HLBS II	Unstandardized Coefficients		Standardized Coefficients	t	*p*	95% CI		Collinearity Statistics	
	B	SH	B			Upper	Lower	Tolerance	VIF
(Constant)	123.484	3.000		41.156	< 0.001				
Physical activity	16.763	3.192	0.312	5.252	< 0.001	10.474	23.052	0.994	1.006
Sharing personal belongings	−32.041	8.789	−0.217	−3.645	< 0.001	−49.359	−14.722	0.993	1.008
Staying in wards for 11–20 people*	9.832	3.355	0.174	2.930	0.004	3.221	16.443	0.994	1.006
Primary education level and below**	−7.738	3.188	−0.144	−2.427	0.016	−14.021	−1.456	0.996	1.004

R^2^ = 0.195; Adjusted R^2^ = 0.181; Durbin-Watson = 2.115; F (4.233) = 13.909; *p* <0.001

* Reference categories: Staying in wards for 1–10 people

** Reference categories: Secondary education

In line with the research questions, the study findings revealed that prisoners had moderate levels of health perception and health-promoting behaviors. The descriptive results addressed the first two research questions. Furthermore, Pearson correlation analyses indicated a statistically significant, positive relationship between health perception and healthy lifestyle behaviors, thereby answering the third question. Lastly, multiple linear regression analyses identified key predictors for both health perception and health-promoting behaviors, responding to the fourth research question.

In the regression analysis conducted by dividing the total scores of the Health-Promoting Lifestyle Profile into quartiles, it was found that health perception significantly increased as the health-promoting lifestyle scores increased (*p* <.05). The group in the highest quartile (Q4) had a health perception score approximately 6.75 points higher than the group in the lowest quartile (Q1) (Table 6).

**Table 6:** Results of multiple linear regression analysis for the prediction of prisoners’ HPS

HPS	Unstandardized Coefficients		Standardized Coefficients	t	*p*	95% CI		Collinearity Statistics	
	B	SH	B			Upper	Lower	Tolerance	VIF
(Fixed)	47.860	0.969		89.433	0.000	45.950	49.769		
HLBS II_4	6.751	1.359	0.384	4.967	0.000	9.428	4.073	0.657	1.522
HLBS II_3	4.207	1.353	0.240	3.108	0.002	6.874	1.540	0.655	1.526
HLBS II_2	3.209	1.365	0.181	2.351	0.020	5.898	0.520	0.659	1.517

R^2^ = 0.099; Adjusted R^2^ = 0.087; Durbin-Watson = 2.128; F (3.233) = 8.436; *p* <.001

## DISCUSSION

This study investigated the health perceptions and health-promoting behaviors of prisoners, the relationship between these variables, and the predictors affecting them. The findings showed that the mean score on the health perception scale was 51.43 ± 7.66, and the mean score on the healthy lifestyle behaviors scale was 130.41 ± 26.90. A statistically significant positive correlation was found between the two variables. In addition, sociodemographic characteristics such as education level and certain health behaviors including physical activity and personal hygiene practices were identified as significant predictors of both health perceptions and health-promoting behaviors.

In this study, health perception and healthy living behaviors of prisoners with primary education and below were found to be lower compared to prisoners with higher education level. According to the results of regression analysis, having primary education level or less decreases health perception of prisoners by 2.085 points and healthy life behaviors by 7.738 points. In previous studies, it was found that the health perception of prisoners improved as the education level increased [9] and low education level was associated with low health perception [8]. Furthermore, it has been shown that there is a positive relationship between educational level and health perception [28] and that prisoners with higher educational level have a better health perception [21]. This may be explained by the fact that individuals with higher education levels often have better socio-economic status prior to incarceration, which facilitates easier access to health and social services as well as health-related information.

In this study, it was determined that the healthy life behaviors of prisoners in wards for 11–20 people were higher than those of prisoners in other wards. According to the results of regression analysis, there was an increase of 9,832 points in the healthy life behaviors of prisoners in these wards. It is also reported in the literature that overcrowding in prisons negatively affects prisoners’ health and self-care behaviors. For example, in a qualitative study, it was reported that overcrowded and cramped prisons negatively affected prisoners’ self-care abilities [29]. Moreover, overcrowding has been shown to lead to a variety of negative effects, such as malnutrition, lack of hygiene, security issues, and difficulties in accessing healthcare [30]. Moreover, overcrowding has also been reported to be a significant source of stress on prisoners’ mental health [31]. In line with these results, as the number of individuals staying in prison wards increases, the healthy living behaviors of prisoners decrease. This may be attributed to several interrelated factors. Overcrowded environments limit personal space and privacy, which can reduce inmates’ motivation to engage in health-promoting behaviors and personal hygiene practices. These conditions often lead to restricted access to clean water, sanitary facilities, and opportunities for physical activity, all of which are essential for maintaining health. Additionally, the increased interpersonal tension and noise in overcrowded wards can elevate stress levels and diminish psychological well-being, making it more difficult for inmates to sustain healthy routines. Therefore, it is thought that reducing the number of prisoners staying in the ward will increase the healthy living behaviors of prisoners.

In this study, it was determined that both health perceptions and healthy life behaviors of prisoners who engaged in regular physical activity were higher than prisoners who did not engage in regular physical activity. According to the results of regression analysis, it was determined that prisoners who engaged in regular physical activity increased their health perception by 2.314 points and their healthy life behaviors by 16.763 points. The literature also shows that regular physical activity significantly improves prisoners’ perception of health. For example, in one study, it was determined that participation in sports activities such as athletics, badminton, table tennis and volleyball positively affected the physical health perception of prisoners and regular participation increased health perception [32]. Furthermore, a study in Israel reported that although 8 out of 10 prisoners did not meet the recommended level of 150 min of physical activity per week, prisoners who participated in any health promotion activity were more likely to reach the recommended activity level and have a better health status [18]. These findings highlight the importance of developing programs that promote regular physical activity in prisons. In particular, increasing access to sports activities and organizing group activities that encourage regular exercise can improve prisoners’ perception of health and healthy living behaviors.

In this study, the health perception of non-smokers was found to be significantly higher than that of smokers. According to the regression analysis, smoking was associated with a 3.329-point decrease in health perception scores. While this finding aligns with studies indicating that smoking cessation efforts can improve self-perceived health [33]. the literature presents inconsistent results on the relationship between smoking and health perception. For instance, some studies report no statistically significant difference in perceived health based on smoking status among prisoners [8, 34]. These inconsistencies may be due to several factors. These inconsistencies may be due to variations in sample characteristics and institutional environments. Recent international studies indicate that smoking is consistently linked to poorer self-rated health. For instance, a study in England found exclusive e-cigarette users had significantly better self-rated health than traditional cigarette smokers [35]. A qualitative study of smoking abstinence after release from smokefree prisons highlighted the health-awareness dimension and motivations tied to perceived health post-incarceration [36]. Thus, our finding—that non-smokers report about 3.3 points higher health perception—parallels trends in both general and emerging carceral literature [37]. However, certain conditions specific to prison environments—like limited access to health education, the normalization of smoking, and lack of support for quitting—may weaken the relationship between smoking and perceived health. These factors might explain why some studies in prison settings do not find a strong or significant link between the two.

The higher health perception of nonsmoking prisoners may be a result of awareness of the negative effects of smoking on health and efforts to protect health. Therefore, further research on prisoners is needed to gain a deeper understanding of the relationship between smoking and health perception.

In this study, no statistically significant difference was found in healthy lifestyle behaviors based on the smoking and healthy nutrition status of prisoners. In the literature, no studies were found that directly examined healthy lifestyle behaviors in relation to these variables specifically within prison populations. One possible explanation for the absence of significant associations in this study may be the subjective and self-reported nature of the “smoking” and “healthy eating” status variables, which were assessed using single yes/no questions. Such dichotomous variables may oversimplify complex behaviors and fail to capture variations in intensity, frequency, or quality of these health-related practices. These methodological limitations suggest that a more nuanced and multidimensional assessment of smoking and nutrition habits may be necessary to better detect associations with healthy lifestyle behaviors in future research.

In this study, both health perception and healthy life behaviors of prisoners who brush their teeth regularly were higher than prisoners who do not brush their teeth regularly. In a study evaluating the effect of a program aimed at improving oral health, it was reported that the number of prisoners who consumed sugary drinks and cigarettes decreased and general oral health status improved during the program [38]. Regular tooth brushing is one of the healthy lifestyle behaviors. It can be said that the health perception and healthy life behaviors of prisoners who brush their teeth regularly increase.

In this study, the health perceptions and healthy life behaviors of the prisoners who did not share personal belongings were higher than the prisoners who shared personal belongings. According to the results of regression analysis, there was a decrease of 6.016 points in health perceptions and 32.041 points in healthy life behaviors of prisoners in case of shared use of personal belongings. This finding shows that the living conditions of prisoners have a significant impact on their health perceptions and healthy living behaviors. The use of communal items may reduce hygiene standards and limit personal space, which may negatively affect prisoners’ perception of health. Moreover, shared belongings can increase the risk of spreading infectious diseases, which can negatively affect health perception and healthy living behaviors. On the other hand, prisoners who do not share personal belongings may be more likely to take care of their health and adopt healthy living behaviors, as they may have more hygienic conditions and personal space. Although the physical environment in prisons such as overcrowded wards, poor sanitation, and lack of ventilation presents significant barriers to practicing health behaviors, it does not prevent individuals from forming perceptions about their health. Prisoners are still aware of their bodily needs, symptoms, discomforts, and personal health goals, even if they cannot fully act on them. These perceptions influence how they prioritize health within the constraints of their environment. Understanding these perceptions is critical, because they provide a foundation for future behavioral changes or for the development of targeted interventions that are realistic and adapted to prison conditions.

### Limitations of the study

Since the research was conducted only in open prisons in one province of Turkey and with male prisoners, the findings cannot be generalized to other prisons and female prisoners.

## CONCLUSIONS

This study demonstrated that prisoners’ perceived health and healthy living behaviors are influenced by several key factors, including ward size, educational level, smoking status, physical activity participation, and personal hygiene practices. Specifically, healthier behaviors were more common among individuals staying in less crowded wards, those with higher educational attainment, and those who engaged in regular physical activity. Conversely, smoking was associated with lower health perception, while consistent hygiene practices—such as tooth brushing, regular showering, and avoiding the shared use of personal items—supported positive health behaviors.

In light of these findings, health interventions in correctional facilities should focus on promoting healthy lifestyle habits and addressing environmental conditions that hinder health. Policy recommendations include:

- Reducing overcrowding in wards to support healthier behavior patterns,
- Implementing regular health education programs that emphasize the importance of hygiene and physical activity,
- Establishing smoking cessation initiatives tailored to the prison population, and.
- Ensuring access to preventive healthcare services through routine and structured nursing interventions.

Improving health conditions in correctional settings not only benefits individual well-being but also contributes to broader public health by facilitating the reintegration of healthier individuals into society.

## Data Availability

Data is available on request from the authors.

## Abbreviations

HLBS-II: Healthy LifestyleBehaviors Scale II
HPS: Health Perception Scale
SPSS: Statistical Package for The Social Sciences
